# Fourier transform and near infrared dataset of dialdehyde celluloses used to determine the degree of oxidation with chemometric analysis

**DOI:** 10.1016/j.dib.2021.107757

**Published:** 2021-12-23

**Authors:** Jonas Simon, Otgontuul Tsetsgee, Nohman Arshad Iqbal, Janak Sapkota, Matti Ristolainen, Thomas Rosenau, Antje Potthast

**Affiliations:** a Department of Chemistry, Institute of Chemistry of Renewable Resources, University of Natural Resources and Life Sciences Vienna (BOKU), Konrad-Lorenz-Strasse 24, Tulln 3430, Austria; b Department of Chemistry, Faculty of Sciences and Engineering, Sorbonne University, Campus Pierre et Marie Curie, 4 place Jussieu, Paris 75005, France; c NE Research Center, UPM Pulp Research and Innovations, Lappeenranta 53200, Finland

**Keywords:** Periodate oxidation, Cellulose chemistry, Multivariate calibration modelling, Partial least-squares regression, Infrared spectroscopy, Chemometrics

## Abstract

This dataset is related to the research article entitled ``A fast method to measure the degree of oxidation of dialdehyde celluloses using multivariate calibration and infrared spectroscopy''. In this article, 74 dialdehyde cellulose samples with different degrees of oxidation were prepared by periodate oxidation and analysed by Fourier-transform infrared (FTIR) and near-infrared spectroscopy (NIR). The corresponding degrees of oxidation were determined indirectly by periodate consumption using UV spectroscopy at 222 nm and by the quantitative reaction with hydroxylamine hydrochloride followed by potentiometric titration. Partial least squares regression (PLSR) was used to correlate the infrared data with the corresponding degree of oxidation (DO). The developed NIR/PLSR and FTIR/PLSR models can easily be implemented in other laboratories to quickly and reliably predict the degree of oxidation of dialdehyde celluloses.

## Specifications Table

**Table:** 

Subject	Chemistry and Chemometrics
Specific subject area	Pulp chemistry and carbohydrate polymers
Type of data	Tables, spectroscopic data and Opus files
How the data were acquired	(1) Infrared (IR) spectra: ‐ NIR: MPA Multi-Purpose Analyzer (Bruker, Billerica, MA) with a fibre optic probe and a Te-InGaAs detector (10 kHz) ‐ FTIR: Frontier FTIR spectrophotometer (PerkinElmer, Waltham, MA, USA) (2) Degree of oxidation (DO): ‐ UV/Vis method: The DO was calculated from the periodate consumption using a LAMBDA 35 UV/Vis spectrometer (PerkinElmer, Waltham, MA) at 222 nm. ‐ Titration method: The DO was determined by the quantitative reaction of the DAC samples with hydroxylamine hydrochloride followed by titration to the initial pH using an 877 Titrino plus instrument (Metrohm AG, Herisau, Switzerland). (3) Partial Least Squares Regression (PLSR): ‐ OPUS QUANT2 package (Bruker Optics, v. 8.2.28)
Data format	Raw (.csv, .o) and analysed Opus files (.q2)
Description of data collection	DAC samples with different degrees of oxidation were generated by periodate oxidation of softwood kraft pulp. The isolated samples were air-dried and analysed using NIR and FTIR spectroscopy. The infrared data were pre-processed using min–max normalisation, first derivative plus multiplicative scattering correction or first derivative plus vector normalisation. The DO of each sample was determined by the two most used methods, the UV/Vis method [1] and the titration or oxime method [2].
Data source location	Institute of Chemistry of Renewable Resources, University of Natural Resources and Life Sciences Vienna (BOKU), Konrad-Lorenz-Strasse 24, 3430 Tulln, Austria
Data accessibility	(1) Infrared (IR) spectra: NIR and FTIR data are available in Mendeley repository data. (2) Degree of oxidation (DO): Data is with this article (Table 2). (3) Partial Least Squares Regression (PLSR): PLSR models processed with OPUS QUANT2 are available in Mendeley repository data and parameters used are with this article (Table 1). https://data.mendeley.com/datasets/bncy3n34v7/draft?a=b69c69fa-86f3-4ce3-916d-87f4c9e90ef9
Related research article	J. Simon, O. Tsetsgee, N. A. Iqbal, J. Sapkota, M. Ristolainen, T. Rosenau, A. Potthast, A fast method to measure the degree of oxidation of dialdehyde celluloses using multivariate calibration and infrared spectroscopy, Carbohydrate Polymers, 10.1016/j.carbpol.2021.118887. [3]

## Value of the Data

• The data can be used to predict the degree of oxidation rapidly and reliably in dialdehyde celluloses.
• Determining the aldehyde content is crucial for tailoring the properties of dialdehyde cellulose, which is applied in areas such as drug delivery [4], [5], [6], medical applications [7], [8], [9], sensor technologies [10], [11], [12] and material science [13].
• This dataset allows researchers to implement this method in everyday research saving money, time and resources.

## 1 Data Description

**Fig. 1:**
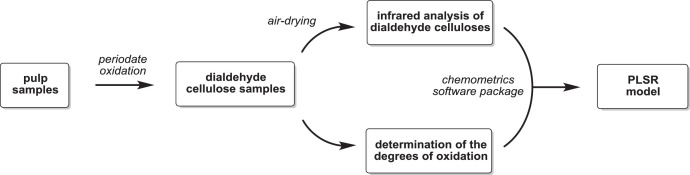
Schematic of the experimental design to collect and analyse the dataset.

**Fig. 2:**
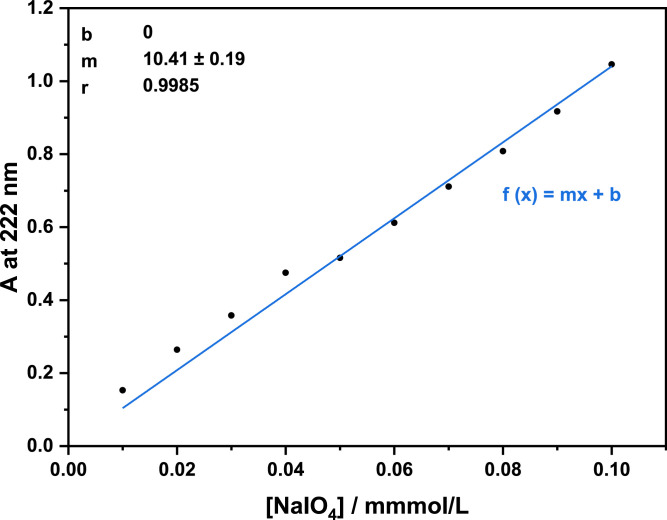
Calibration curve for the determination of periodate concentration by ultraviolet–visible spectroscopy at 222 nm.

All data refer to the original research article ``A fast method to measure the degree of oxidation of dialdehyde celluloses using multivariate calibration and infrared spectroscopy'' [3]. Fig. 1 shows a schematic of the experimental design to collect and analyse the dataset. The data in Table 2 displays the isolated dialdehyde cellulose samples with their file names (NIR and FTIR dataset) and their obtained degrees of oxidation (DO)—from periodate consumption using UV/Vis spectroscopy (DO_UV/Vis_) and from potentiometric titration after quantitative reaction with hydroxylamine hydrochloride (DO_Titration_). The degrees of oxidation from periodate consumption are calculated using a calibration curve (Fig. 2). The corresponding spectral raw data is available in Mendeley repository data ("Dataset">" raw_data": Spectral raw data for each PLSR model, .csv files). The isolated DAC samples were used to construct four PLSR models that correlate the NIR and FTIR data with the corresponding DO. Table 1 summarizes the parameters of partial least-squares regression. OPUS QUANT2 was used to develop the NIR/PLSR models (1 and 2) and FTIR/PLSR models (3 and 4), which are available in Mendeley repository data ("Dataset">" processed_data": OPUS files for each model with the corresponding spectra, .q2 and .o files).

**Table 1:** Parameters of partial least-squares regression models (1–4).

	Model 1	Model 2	Model 3	Model 4
	NIR/PLSR	NIR/PLSR	FTIR/PLSR	FTIR/PLSR
**DO measured by**	Potentiometric titration (DO_Titration_)	UV/Vis spectroscopy (DO_UV/Vis_)	Potentiometric titration (DO_Titration_)	UV/Vis spectroscopy (DO_UV/Vis_)
**Range / cm^−1^**	9000 to 4000	9000 to 6500	4000 to 650	4000 to 650
**Pre-processing**	Min-Max normalization	First derivative + multiplicative scatter correction	First derivative + multiplicative scatter correction	First derivative + vector normailsation
**Latent variable**	9	8	3	10

**Table 2:** Overview of dialdehyde cellulose samples (from SWKP) analysed with their degrees of oxidation (DO) obtained from the periodate consumption by UV/Vis spectroscopy (DO_UV/Vis_) and potentiometric titration (DO_Titration_).

Sample		FTIR *file name*	NIR *file name*	DO_UV/Vis_ /%	DO_Titration_ /%
KP-2–1	–3	KP-2–1-3A KP-2–1-3B KP-2–1-3C	KP-2–1-3 KP-2–1-3.1 KP-2–1-3.2	*no sample was taken*	14.16 (±0.00)
	–4	*not acquired*	KP-2–1-4 KP-2–1-4.1 KP-2–1-4.2	*no sample was taken*	15.44 (±2.01)
	–10	KP-2–1-10a KP-2–1-10b KP-2–1-10c	KP-2–1-10 KP-2–1-10.1 KP-2–1-10.2	*no sample was taken*	39.43 (±0.13)
	–11	KP-2–1-11a KP-2–1-11b KP-2–1-11c	KP-2–1-11 KP-2–1-11.1 KP-2–1-11.2	*no sample was taken*	47.15 (±1.49)
	–12	KP-2–1-12a KP-2–1-12b KP-2–1-12c	KP-2–1-12 KP-2–1-12.1 KP-2–1-12.2	*no sample was taken*	49.51 (±0.35)
	–15	KP-2–1-15a KP-2–1-15b KP-2–1-15c	KP-2–1-15 KP-2–1-15.1 KP-2–1-15.2	*no sample was taken*	59.14 (±0.14)

KP-2–2	–2	KP-2–2-2a KP-2–2-2b KP-2–2-2c	KP-2–2-2 KP-2–2-2.1 KP-2–2-2.2	*measurement failed*	13.25 (±0.33)
	–3	KP-2–2-3a KP-2–2-3b KP-2–2-3c	KP-2–2-3 KP-2–2-3.1 KP-2–2-3.2	17.43 (±0.62)	*sample size was too small*
	–4	KP-2–2-4a KP-2–2-4b KP-2–2-4c	KP-2–2-4 KP-2–2-4.1 KP-2–2-4.2	12.40 (±1.62)	*sample size was too small*
	–5	KP-2–2-5a KP-2–2-5b KP-2–2-5c	KP-2–2-5 KP-2–2-5.1 KP-2–2-5.2	17.38 (±1.70)	24.01 (±0.21)
	–6	KP-2–2-6a KP-2–2-6b KP-2–2-6c	KP-2–2-6 KP-2–2-6.1 KP-2–2-6.2	20.50 (±0.98)	27.75 (±0.73)
	–7	KP-2–2-7a KP-2–2-7b KP-2–2-7c	KP-2–2-7 KP-2–2-7.1 KP-2–2-7.2	28.06 (±4.22)	*sample size was too small*
	–8	KP-2–2-8a KP-2–2-8b KP-2–2-8c	KP-2–2-8 KP-2–2-8.1 KP-2–2-8.2	32.24 (±0.34)	35.25 (±0.06)
	–9	KP-2–2-9a KP-2–2-9b KP-2–2-9c	KP-2–2-9 KP-2–2-9.1 KP-2–2-9.2	38.67 (±0.38)	39.00 (±1.20)
	–10	KP-2–2-10a KP-2–2-10b KP-2–2-10c	KP-2–2-10 KP-2–2-10.1 KP-2–2-10.2	40.98 (±1.80)	45.00 (±1.05)
	–11	KP-2–2-11a KP-2–2-11b KP-2–2-11c	KP-2–2-11 KP-2–2-11.1 KP-2–2-11.2	42.35 (±0.79)	46.22 (±0.76)
	–12	KP-2–2-12a KP-2–2-12b KP-2–2-12c	KP-2–2-12 KP-2–2-12.1 KP-2–2-12.2	44.14 (±1.42)	49.75 (±0.82)
	–13	KP-2–2-13a KP-2–2-13b KP-2–2-13c	KP-2–2-13 KP-2–2-13.1 KP-2–2-13.2	50.37 (±1.18)	53.02 (±0.73)
	–14	KP-2–2-14a KP-2–2-14b KP-2–2-14c	KP-2–2-14 KP-2–2-14.1 KP-2–2-14.2	50.77 (±1.40)	55.45 (±0.58)
	–15	KP-2–2-15a KP-2–2-15b KP-2–2-15c	KP-2–2-15 KP-2–2-15.1 KP-2–2-15.2	53.03 (±0.83)	55.48 (±0.93)
JS-22	–1	JS-22–1A JS-22–1B JS-22–1C	JS-22–1 JS-22–1.1 JS-22–1.2	74.27 (±1.00)	85.91 (±1.56)
	–2	JS-22–2A JS-22–2B JS-22–2C	JS-22–2 JS-22–2.1 JS-22–2.2	75.97 (±0.57)	77.69 (±0.60)
	–3	JS-22–3A JS-22–3B JS-22–3C	JS-22–3 JS-22–3.1 JS-22–3.2	63.34 (±0.81)	74.98 (±0.18)
	-5	JS-22–5A JS-22–5B JS-22–5C	JS-22–5 JS-22–5.1 JS-22–5.2	65.67 (±0.07)	70.62 (±2.81)
	–6	JS-22–6A JS-22–6B JS-22–6C	JS-22–6 JS-22–6.1 JS-22–6.2	45.51 (±0.06)	46.66 (±0.47)

JS-23	–3	JS-23–3A JS-23–3B JS-23–3C	JS-23–3 JS-23–3.1 JS-23–3.2	*no sample was taken*	11.16 (±0.98)
	–4	JS-23–4A JS-23–4B JS-23–4C	JS-23–4 JS-23–4.1 JS-23–4.2	*no sample was taken*	8.37 (±1.83)
	–6	JS-23–6A JS-23–6B JS-23–6C	JS-23–6 JS-23–6.1 JS-23–6.2	*no sample was taken*	16.30 (±1.29)

KP-1–1.2		KP-1–1.2-a KP-1–1.2-b KP-1–1.2-c	*not acquired*	56.54 (±2.23)	*sample size was too small*

KP-1–2.1		KP-1–2.1-a KP-1–2.1-b KP-1–2.1-c	*not acquired*	27.97 (±1.32)	*sample size was too small*

KP-2–4	–1	KP-2–4-1a KP-2–4-1b KP-2–4-1c	KP-2–4-1 KP-2–4-1.1 KP-2–4-1.2	12.92 (±1.39)	7.72 (±1.40)
	–2	KP-2–4-2a KP-2–4-2b KP-2–4-2c	KP-2–4-2 KP-2–4-2.1 KP-2–4-2.2	12.94 (±1.79)	*sample size was too small*
	–4	KP-2–4-4a KP-2–4-4b KP-2–4-4c	KP-2–4-4 KP-2–4-4.1 KP-2–4-4.2	22.22 (±1.21)	12.25 (±0.07)
	–5	KP-2–4-5a KP-2–4-5b KP-2–4-5c	KP-2–4-5 KP-2–4-5.1 KP-2–4-5.2	21.34 (±1.63)	13.88 (±0.60)
	–6	KP-2–4-6a KP-2–4-6b KP-2–4-6c	KP-2–4-6 KP-2–4-6.1 KP-2–4-6.2	25.34 (±0.98)	15.49 (±0.49)
	–7	KP-2–4-7a KP-2–4-7b KP-2–4-7c	KP-2–4-7 KP-2–4-7.1 KP-2–4-7.2	22.99 (±0.60)	15.85 (±0.73)
	–8	KP-2–4-8a KP-2–4-8b KP-2–4-8c	KP-2–4-8 KP-2–4-8.1 KP-2–4-8.2	27.27 (±1.69)	17.17 (±0.25)
	–9	KP-2–4-9a KP-2–4-9b KP-2–4-9c	KP-2–4-9 KP-2–4-9.1 KP-2–4-9.2	24.01 (±1.49)	*sample size was too small*
	–10	KP-2–4-10a KP-2–4-10b KP-2–4-10c	KP-2–4-10 KP-2–4-10.1 KP-2–4-10.2	28.41 (±0.91)	19.51 (±0.11)
	–11	KP-2–4-11a KP-2–4-11b KP-2–4-11c	KP-2–4-11 KP-2–4-11.1 KP-2–4-11.2	27.89 (±1.36)	21.80 (±0.21)
	–12	KP-2–4-12a KP-2–4-12b KP-2–4-12c	KP-2–4-12 KP-2–4-12.1 KP-2–4-12.2	31.20 (±1.55)	22.23 (±0.69)
	–13	KP-2–4-13a KP-2–4-13b KP-2–4-13c	KP-2–4-13 KP-2–4-13.1 KP-2–4-13.2	33.80 (±0.10)	24.07 (±0.21)
	–14	KP-2–4-14a KP-2–4-14b KP-2–4-14c	KP-2–4-14 KP-2–4-14.1 KP-2–4-14.2	36.73 (±0.16)	*sample size was too small*
	–15	KP-2–4-15a KP-2–4-15b KP-2–4-15c	KP-2–4-15 KP-2–4-15.1 KP-2–4-15.2	37.14 (±0.27)	26.03 (±1.56)

KP-1–2.2-redo		KP-1–2.2-redo-a KP-1–2.2-redo-b KP-1–2.2-redo-c	*not acquired*	69.80 (±1.70)	*sample size was too small*

KP-2–3	–1	KP-2–3-1a KP-2–3-1b KP-2–3-1c	KP-2–3-1 KP-2–3-1.1 KP-2–3-1.2	10.18 (±0.18)	6.54 (±0.44)
	–2	KP-2–3-2a KP-2–3-2b KP-2–3-2c	KP-2–3-2 KP-2–3-2.1 KP-2–3-2.2	21.83 (±0.90)	9.46 (±0.37)
	–3	KP-2–3-3a KP-2–3-3b KP-2–3-3c	KP-2–3-3 KP-2–3-3.1 KP-2–3-3.2	15.14 (±1.35)	13.49 (±0.75)
	–4	KP-2–3-4a KP-2–3-4b KP-2–3-4c	KP-2–3-4 KP-2–3-4.1 KP-2–3-4.2	18.16 (±2.28)	*sample size was too small*
	–5	KP-2–3-5a KP-2–3-5b KP-2–3-5c	KP-2–3-5 KP-2–3-5.1 KP-2–3-5.2	21.53 (±0.43)	20.56 (±2.86)
	–6	KP-2–3-6a KP-2–3-6b KP-2–3-6c	KP-2–3-6 KP-2–3-6.1 KP-2–3-6.2	21.46 (±0.97)	20.94 (±0.20)
	–7	KP-2–3-7a KP-2–3-7b KP-2–3-7c	KP-2–3-7 KP-2–3-7.1 KP-2–3-7.2	26.47 (±0.64)	22.18 (±0.49)
	–8	KP-2–3-8a KP-2–3-8b KP-2–3-8c	KP-2–3-8 KP-2–3-8.1 KP-2–3-8.2	30.01 (±0.59)	*sample size was too small*
	–9	KP-2–3-9a KP-2–3-9b KP-2–3-9c	KP-2–3-9 KP-2–3-9.1 KP-2–3-9.2	33.89 (±0.51)	27.91 (±0.50)
	–10	KP-2–3-10a KP-2–3-10b KP-2–3-10c	KP-2–3-10 KP-2–3-10.1 KP-2–3-10.2	*measurement failed*	29.07 (±0.59)
	–11	KP-2–3-11a KP-2–3-11b KP-2–3-11c	KP-2–3-11 KP-2–3-11.1 KP-2–3-11.2	33.91 (±0.31)	32.27 (±0.14)
	–12	KP-2–3-12a KP-2–3-12b KP-2–3-12c	KP-2–3-12 KP-2–3-12.1 KP-2–3-12.2	39.62 (±1.13)	33.55 (±1.91)
	–13	KP-2–3-13a KP-2–3-13b KP-2–3-13c	KP-2–3-13 KP-2–3-13.1 KP-2–3-13.2	43.31 (±0.98)	37.00 (±0.93)
	–14	KP-2–3-14a KP-2–3-14b KP-2–3-14c	KP-2–3-14 KP-2–3-14.1 KP-2–3-14.2	43.21 (±1.77)	39.29 (±0.54)
	–15	KP-2–3-15a KP-2–3-15b KP-2–3-15c	KP-2–3-15 KP-2–3-15.1 KP-2–3-15.2	47.97 (±0.99)	42.21 (±0.16)

JS-20	–1	JS-20–1A JS-20–1B JS-20–1C	JS-20–1 JS-20–1.1 JS-20–1.2	5.76 (±0.40)	4.96 (±0.37)
	–2	JS-20–2A JS-20–2B JS-20–2C	JS-20–2 JS-20–2.1 JS-20–2.2	7.94 (±1.39)	*sample size was too small*
	–3	JS-20–3A JS-20–3B JS-20–3C	JS-20–3 JS-20–3.1 JS-20–3.2	9.44 (±0.70)	5.61 (±0.75)
	–4	JS-20–4A JS-20–4B JS-20–4C	JS-20–4 JS-20–4.1 JS-20–4.2	11.87 (±0.11)	5.91 (±0.27)
	–5	JS-20–5A JS-20–5B JS-20–5C	JS-20–5 JS-20–5.1 JS-20–5.2	12.55 (±0.34)	3.84 (±0.50)
	–6	JS-20–6A JS-20–6B JS-20–6C	JS-20–6 JS-20–6.1 JS-20–6.2	14.43 (±0.62)	8.63 (±0.42)
	–7	JS-20–7A JS-20–7B JS-20–7C	JS-20–7 JS-20–7.1 JS-20–7.2	14.83 (±0.40)	10.36 (±0.35)
	–8	JS-20–8A JS-20–8B JS-20–8C	JS-20–8 JS-20–8.1 JS-20–8.2	14.45 (±1.90)	11.01 (±0.72)
	–9	JS-20–9A JS-20–9B JS-20–9C	JS-20–9 JS-20–9.1 JS-20–9.2	16.26 (±0.50)	11.85 (±0.16)
	–10	JS-20–10A JS-20–10B JS-20–10C	JS-20–10 JS-20–10.1 JS-20–10.2	18.22 (±0.45)	12.53 (±0.44)
	–11	JS-20–11A JS-20–11B JS-20–11C	JS-20–11 JS-20–11.1 JS-20–11.2	19.15 (±0.03)	14.14 (±0.22)
	–12	JS-20–12A JS-20–12B JS-20–12C	JS-20–12 JS-20–12.1 JS-20–12.2	18.16 (±1.45)	*sample size was too small*
	–13	JS-20–13A JS-20–13B JS-20–13C	JS-20–13 JS-20–13.1 JS-20–13.2	19.57 (±0.51)	17.14 (±1.03)

https://data.mendeley.com/datasets/bncy3n34v7/draft?a=b69c69fa-86f3-4ce3-916d-87f4c9e90ef9

## 2 Experimental Design, Materials and Methods

The dataset was generated by first oxidising pulp samples with sodium periodate. After that, the isolated samples were air-dried and analysed by NIR and FTIR spectroscopy. The correlating degrees of oxidation were determined by UV/Vis spectroscopy and potentiometric titration after hydroxylamine hydrochloride treatment. Finally, the IR datasets were correlated to the corresponding degrees of oxidation using the OPUS QUANT2 package (Bruker Optics, v. 8.2.28). The provided data can be used to reproduce the PLSR models with any chemometrics software package or use the analysed Opus files to predict the DO in any periodate oxidized cellulose sample. The following sections are expanded versions of the description of the methods presented in our previous works [3].

### 2.1 Chemicals and reagents

UPM-Kymmene Oyj (Lappeenranta, Finland) provided softwood kraft pulp samples used as the starting material in periodate oxidation. Sodium periodate (≥99.8%; Sigma Aldrich; oxidant in the oxidation of pulp to dialdehyde celluloses) and hydroxylamine hydrochloride (99%; Sigma Aldrich) were used without further purification.

### 2.2 Periodate oxidation to prepare dialdehyde cellulose samples

DAC samples were prepared by periodate oxidation of softwood kraft pulp. Air-dried softwood kraft pulp was disintegrated in deionised water using a commercial kitchen blender (3 times for 10 s). It was then filtered and added to a sodium periodate solution. The flask was covered with aluminum foil to limit side reactions (i.e., degradation of sodium periodate). The temperature (room temperature to 50°C), periodate concentration (0.8 eq to 2 eq) and reaction duration (up to 3 days) were varied to prepare DAC samples with degrees of oxidation between 0 and 80%. The isolated DAC samples were thoroughly washed with water (2–3x) and ethanol (1x) using vacuum filtration.

### 2.3 Infrared measurements

Before recording the IR spectra, the DAC samples were air-dried for 2 to 14 days and conditioned in the measuring room before analysis. Other drying techniques (such as oven drying or freeze-drying) are not recommended since a controlled equilibrium between the free aldehyde and its masked forms is needed. All isolated DAC samples were measured three times with NIR and FTIR spectroscopy. The total number of spectra slightly varies since single measurements failed or the sample size was too small for NIR analysis (Table 1). The NIR spectra were recorded using an MPA Multi-Purpose Analyzer (Bruker, Billerica, MA) with a fibre optic probe and a Te-InGaAs detector (10 kHz). The parameters for all analyses included an 8 cm^−1^ resolution, the 12,500–4000 cm^−1^ spectral range and 32 scans per sample. All measurements were conducted at room temperature using aluminum foil as the background. The fibre optic probe was pressed onto three different (randomly chosen) positions of the DAC surface to consider inhomogeneity within the sample. The FTIR spectra were recorded using a Frontier FTIR spectrophotometer (PerkinElmer, Waltham, MA, USA) in conjunction with the attenuated total reflection (ATR) technique. All analyses' parameters include a resolution of 4cm^−1^, the spectral range of 4000–650 cm^−1^, and 64 scans per sample. All infrared measurements were conducted at room temperature. The three-fold measurements were conducted at three different (randomly chosen) positions of the DAC sample to consider inhomogeneity within the sample.

### 2.4 Determining the degree of oxidation of isolated dialdehyde samples

The degrees of oxidation were determined from the periodate consumption through UV/Vis spectroscopy [1] and potentiometric titration after the quantitative reaction with hydroxylamine hydrochloride [2].

For potentiometric analysis, 18–22 mg of the isolated dialdehyde celluloses were freeze-dried and shaken in 5 mL of 0.25 M hydroxylamine hydrochloride solution for 48 h. The hydroxylamine hydrochloride solution was adjusted to pH 4.6. Hydroxylamine hydrochloride quantitively reacts with the carbonyl groups of DAC, releasing one mole of hydrochloric acid per aldehyde functionality. For each sample, 2.00 mL were diluted with 5 mL of deionized water to ensure sufficient contact with the pH electrode. Each sample was prepared in duplicate and titrated back to pH 4.6 with 0.01 M sodium hydroxide solution. The DO was then calculated from the volume of consumed sodium hydroxide $V_{NaOH}$ according to

(1) $$DO_{Titration}\left[\%\right]=\frac{V_{NaOH}\cdot \left[NaOH\right]\cdot M_{AGU}\cdot V_{1}}{2\cdot m_{DAC,0}\cdot V_{0}}\cdot 100-DO_{blank}$$

where [NaOH] is the NaOH concentration, $M_{AGU}$ the molecular weight of the anhydroglucose unit, $m_{DAC,0}$ the weight of the freeze-dried DAC, $V_{0}$ the initial volume of the added hydroxylamine hydrochloride, $V_{1}$ the volume of the titrated oxime solution, and $DO_{blank}$ the DO of the unreacted pulp as a blank.

The DO was also determined from the periodate consumption by UV/Vis spectroscopy at 222 nm (DO_UV/Vis_). 100 μL of each filtrate was diluted with deionized water, and the remaining periodate concentration was calculated from the periodate absorbance at 222 nm. The dilution factor was varied depending on the equivalents of sodium periodate used to measure absorbances in the range of 0.5 to 1.1. UV/Vis measurements were performed using a LAMBDA 35 UV/Vis spectrometer (PerkinElmer, Waltham, MA). The UV/Vis spectrometer was referenced to deionised water using a quartz cuvette with a 10 mm path length. Assuming no side reactions, the DO was calculated according to

(2) $$DO_{UV/Vis}\left[\%\right]=\frac{n_{OH,consumed}}{n_{pulp,0}}\cdot 100=\frac{M_{AGU}\cdot \left[\frac{m_{NaIO_{4},0}}{M_{NaIO_{4}}}-\left(\frac{A}{b\;}\cdot F_{D}\cdot V\right)\right]}{m_{pulp,0}}\cdot 100$$

where $m_{NaIO_{4},0}$ and $m_{pulp,0}$ are the mass of sodium periodate and pulp, respectively; $M_{NaIO_{4}}$ is the molecular weight of the sodium periodate, A the arithmetic mean of the measured absorbance, b the calibration curve slope (Fig. 2), $F_{D}$ the dilution factor, and V the solvent (deionized water) volume; $M_{AGU}$ is the molecular weight of the anhydroglucose unit (AGU), simplified on the assumption that the pulp consists of cellulose only.

### 2.5 Partial least squares regression

The unprocessed NIR and FTIR data were pre-processed using min–max normalisation, first derivative plus multiplicative scattering correction or first derivative plus vector normalisation. The PLSR models were calculated using the OPUS QUANT2 package (Bruker Optics, v. 8.2.28; parameters in Table 1). The PLSR algorithm automatically validated the obtained correlation model with a selected test set of the recorded IR spectra. In addition, the PLS 1 algorithm in OPUS QUANT2 was used to determine the best pre-processing method and the optimal spectral range (Table 1). Leave-one-out cross-validation was used. Two sets of infrared data (NIR and FTIR) with two different degrees of oxidation (from the periodate consumption and potentiometric titration) give four PLSR models, which are all available in Mendeley repository data.

## CRediT authorship contribution statement

**Jonas Simon:** Conceptualization, Visualization, Data curation, Formal analysis, Writing – original draft, Writing – review & editing. **Otgontuul Tsetsgee:** Data curation, Formal analysis, Writing – review & editing. **Nohman Arshad Iqbal:** Conceptualization, Data curation, Formal analysis, Writing – review & editing. **Janak Sapkota:** Data curation, Formal analysis, Writing – review & editing. **Matti Ristolainen:** Data curation, Formal analysis, Writing – review & editing. **Thomas Rosenau:** Conceptualization, Visualization, Supervision, Writing – review & editing. **Antje Potthast:** Conceptualization, Visualization, Data curation, Formal analysis, Supervision, Writing – review & editing.

## Declaration of Competing Interest

The authors declare that they have no known competing financial interests or personal relationships that could have appeared to influence the work reported in this paper.
